# Time trends in Alzheimer’s disease mortality attributable to metabolic risks and smoking in China from 1990 to 2019: an age-period-cohort analysis

**DOI:** 10.3389/fnagi.2024.1425577

**Published:** 2024-07-03

**Authors:** Simeng Sun, Ting Zhang, Hao Yu, Ting Xia, Yunan Yao, Mengting Sun, Hongmei Liang, Qiaoyu Huang, Weiwei Wang, Huafeng Yang, Xin Hong

**Affiliations:** ^1^Nanjing Municipal Center for Disease Control and Prevention, Nanjing, Jiangsu, China; ^2^Jiangsu Provincial Center for Disease Control and Prevention, Nanjing, Jiangsu, China; ^3^Monash Addiction Research Centre, Monash University, Clayton, VIC, Australia; ^4^Nantong Center for Disease Control and Prevention, Nantong, Jiangsu, China; ^5^Nanjing Drum Tower Hospital, Nanjing, Jiangsu, China; ^6^Xinhua Hospital Affiliated to Shanghai Jiao Tong University School of Medicine, Shanghai, China

**Keywords:** Alzheimer’s disease, risk factors, metabolic risks, high body mass index, high fasting plasma glucose, smoking, time trends

## Abstract

**Background:**

With the increase in the aging population worldwide, Alzheimer’s disease has become a rapidly increasing public health concern. In the Global Burden of Disease Study 2019, there are three risk factors judged to have evidence for a causal link to Alzheimer’s disease and other dementias: smoking, high body-mass index (HBMI), and high fasting plasma glucose (HFPG).

**Objective:**

This study aimed to analyze trends in AD mortality and the relevant burden across China from 1990 to 2019, as well as their correlation with age, period, and birth cohort.

**Methods:**

The data were extracted from the GBD 2019. Trends in AD mortality attributable to metabolic risks (HFPG and HBMI) and smoking were analyzed using Joinpoint regression. The age-period-cohort (APC) model was used to evaluate cohort and period effects.

**Results:**

From 1990 to 2019, the overall age-standardized mortality rate of AD increased, especially in women. There was an increase in AD mortality due to smoking in the net drift, and it was more significant in women (0.46, 95%CI = [0.09, 0.82]) than men (−0.03, 95%CI = [−0.11, 0.05]). For the cause of HFPG, the net drift values for men and women were 0.82% and 0.43%. For HBMI, the values were 3.14% and 2.76%, respectively, reflecting substantial increases in AD mortality.

**Conclusion:**

Time trends in AD mortality caused by metabolic risks and smoking in China from 1990 to 2019 have consistently increased. Therefore, it is necessary to prevent excessive weight gain and obesity during the later stages of life, especially for females.

## 1 Introduction

As a result of theCheck if the section headers (i.e., section levelling) have been correctly captured. increasing number of old individuals globally, Alzheimer’s disease (AD) has emerged as a growing public health problem (Yan et al., 2019). In 2019, AD was one of the most burdensome neurological conditions in China, with disability-adjusted life years (DALYs) of 189.47 (75.72–453.76). There were 10 million AD patients in China in 2020, and with the rapid growth of an aging population, it is expected to exceed 20 million by 2050 (Yin et al., 2024). According to our recent study, AD’s overall age-standardized mortality rate (ASMR) increased. Between 2000 and 2019, recorded deaths from AD climbed by about 145% (Frick et al., 2023). The COVID-19 pandemic in 2020 and 2021 may have exacerbated AD mortality trends (Alzheimer’s and Dementia, 2023).

The factors contributing to injury and disease, including environmental, behavioral, and metabolic risks, are the key areas where the most effective way to prevent a decline in health is through public health initiatives (Li et al., 2022). Therefore, to improve population health, it is necessary to understand both the injuries and diseases that contribute to the health burden, and the risks that contribute to them (GBD 2017 Risk Factor Collaborators, 2018). Analyzing the extent of risk factors for AD mortality and providing interventions from behavioral and metabolic perspectives is an essential public health measure to reduce health loss effectively. In the Global Burden of Diseases, Injuries, and Risk Factors Study 2019 (The Global Burden of Disease [GBD], 2019), three risk factors were determined to possess substantial evidence supporting a causal connection to AD: smoking, high body mass index (HBMI), and high fasting plasma glucose (HFPG) (Institute for Health Metrics and Evaluation [IHME], 2020). Smoking is commonly acknowledged as a significant contributor to premature morbidity and mortality. However, effectively tracking smoking rates and trends worldwide has proven difficult (GBD 2015 Tobacco Collaborators, 2017). Smoking might indirectly impact the risk factors for several diseases, including diabetes mellitus, coronary heart disease, and other metabolic diseases because smoking causes vasoconstriction, atherogenesis, thrombogenesis, and endothelial dysfunction (Zhou and Wang, 2021). Metabolic factors can affect the course of AD. The progress in metabolomics has revealed the intricate nature of the dynamic changes linked to the evolution of AD, highlighting the challenges in creating effective therapeutic strategies Wilkins (Cheng et al., 2018; Wilkins and Trushina, 2018). Previous epidemiological research suggests that there is a connection between insulin resistance, diabetes, and an increased risk of AD. A longitudinal study noted that HFPG, measured up to four decades before death from AD, is associated with greater concentrations of glucose in brain tissue throughout the brain (Leibson et al., 1997; Ott et al., 1999; Luchsinger et al., 2001; An et al., 2018). Animal experiments have demonstrated that elevated blood glucose levels are associated with increased blood-brain barrier permeability (Rom et al., 2020) and neuronal apoptosis (Russell et al., 1999). High blood glucose can stimulate the production of reactive oxygen species by plasma albumin (Mohanty et al., 2000), induce endothelial dysfunction (Sena et al., 2013), promote systemic inflammation, increase the number of activated microglial cells, and initiate neuroinflammation, consequently impacting cognitive and memory functions (Drake et al., 2011; Wanrooy et al., 2018). Epidemiological studies have shown that patients with diabetes have a 1.3-fold to 5.5-fold increase in risk of developing AD in comparison with healthy individuals. Even among participants without diabetes, the risk of AD and other dementias increased with increasing glucose levels. As accumulating evidence suggests an association between HFPG with AD and other dementias, it is necessary to evaluate the global burden of AD and other dementias attributable to HFPG to identify the vulnerable population and formulate comprehensive prevention strategies to respond to the rapidly growing burden of AD and other dementias. Efforts have been undertaken to explore the variations in AD mortality over time and mortality rates unique to different age groups. Nevertheless, the impact of metabolic risks and smoking on AD mortality to temporal and cohort effects remains unclear (Vancheri et al., 2022). This Article aims to examine time trends in AD mortality caused by metabolic risks and smoking in China. We analyzed pertinent data obtained from GBD 2019 to aid in formulating focused policies and strategies for dementia control, improving public health, and guiding the efficient distribution of medical resources.

## 2 Materials and methods

### 2.1 Data source and study design

The risk factors of AD deaths in China were from the GBD 2019 Study at the national level (GBD 2019 Diseases and Injuries Collaborators, 2020), freely available from the Global Health Data Exchange.^1^ The methodology, statistical techniques, and metrics used in the GBD 2019 project have been documented elsewhere, such as sex- and age-specific annual deaths and ASMR of AD caused by HBMI, HFPG, and smoking. The GBD 2019 encompasses 369 diseases and injuries, 21 regions, and 204 countries and territories (GBD 2019 Demographics Collaborators, 2020; GBD 2019 Risk Factors Collaborators, 2020; Roth et al., 2020). HFPG was defined as fasting plasma glucose above the theoretical minimum-risk exposure level, 4.8–5.4 mmol/L, and HBMI as BMI ≥ 25 kg/m^2^ (The Global Burden of Disease [GBD], 2019; Li et al., 2023).

### 2.2 Alzheimer’s disease identification

According to the cause list mapped to the 10th International Statistical Classification of Diseases and Related Health Problems (ICD-10), the GBD 2019, and the ICD-10 codes (The Lancet Infectious Diseases, 2018; WHO, 2021; Dhana et al., 2022), According to the ICD-10, AD is recognized as G30.0 Early-onset AD (<65 years old) and G30.1 Late-onset AD (>65 years old).

### 2.3 Statistical analyses

To evaluate the extent of changes in mortality due to AD over time, we employed the joinpoint regression model (version 4.9.1). The joinpoint regression model is a collection of linear statistical models used to evaluate the trends in AD mortality attributable to metabolic risks and smoking. This model is a calculating approach to estimate the changing rule of illness rates using the least square method, avoiding the non-objectivity of typical trend analyses based on linear trends. Calculating the square sum of the residual error between the estimated and actual values yields the turning point of the shifting trend. The fundamental concept of joinpoint is to partition a continuous trend line into distinct parts, every characterized by continuous linearity, to assess the disease-changing characteristics unique to distinct time intervals (Kim et al., 2000). Joinpoint (version 4.9.1.0; National Cancer Institute, Rockville, MD, USA) was used to create this model. The joinpoint model allowed us to assess the average annual percent changes (AAPCs) and the related 95% confidence intervals (CIs) (Fu et al., 2023). AAPC was determined by assigning weights to the regression coefficients of annual percent changes (APCs) to measure the trend across the period. The APC uses a log-linear model to compute each segment, with APC = [e(β)−1] × 100%, and the symbol β represents the gradient of the trend segment (Muggeo, 2010). We use a permutation test to determine the related segment significance of APCs; the Joinpoint Regression Programme revealed the best-proposed model. The APC model, grounded on the Poisson distribution, can capture the accumulated effect of health risks. The APC model can be expressed in a general logarithmic linear form:


$$\rho =\alpha_{a}+\beta_{p}+\gamma_{c}$$

where ρ indicates the expected rate, α_*a*_, β_*p*_, and γ_*c*_ indicate the effects of age, period, and cohort of the APC model (Clayton and Schifflers, 1987). In this study, the age effect pertains to variations in AD mortality resulting from physiological and pathological changes occurring naturally with advancing age. The period effects refer to variations in AD mortality resulting from different events that transpire as time goes on. The cohort effects pertain to variations in AD mortality rates resulting from modifications to one’s way of life or exposure to risk factors that differ inter-generationally.

To examine differences in age, time, and cohort concerning the AD mortality trends caused by smoking and metabolic risks, we utilized the APC model provided by the National Cancer Institute^2^ (Rosenberg et al., 2014). This study focused on adults aged 50+. The age groups were separated into eight categories, each spanning 5 years, ranging from 50–54 to 85 years and older. The period was divided into six consecutive phases of 5 years each, starting from 1990 to 1994 and ending in 2015 to 2019. As a result, ten consecutive birth cohorts were created by grouping individuals based on their age and the period they were born in, ranging from the 1905 to 1910 cohort to the 1965–1969 cohort. The logarithmic linear trend, known as the drift, was employed to construct the AAPC for outcome measurements across time, considering both period and birth cohort. The longitudinal age curves were constructed to illustrate the impact of age by computing the estimated rates specific to each age group for the reference cohort (Zhou et al., 2024). The impact of the period and cohort was indicated by the rate ratios (RRs) compared to the reference period and cohort while considering chronological age and the non-linear aspect of the period and cohort (McNally et al., 1997). Local drift values reflected the temporal trend of AD mortality caused by risk factors in each specific age group. A mortality shift was judged substantial if the absolute drift value exceeded 1% (Guo et al., 2023). The Wald chi-squared test was employed to ascertain the statistical significance of the functions; the two-sided *p* < 0.05 means statistical significance (Nikooienejad and Johnson, 2021).

## 3 Results

### 3.1 Trends in AD mortality attributable to metabolic risks and smoking

Table 1 and Supplementary Figures 1, 2 display the trends in AD mortality related to smoking, HFPG, and HBMI. The number of deaths from AD in China increased from 23998 to 93441 over the past three decades, and the mortality rate increased from 2.03 to 6.57%. We conducted a more in-depth analysis of the ASMR of AD caused by smoking, HFPG, and HBMI. The number of deaths from AD attributable to smoking increased by 2.5-fold between 1990 and 2019 (from 15782 to 55531), and ASMR increased in both men (AAPC = 0.37%, 95%CI = [0.25, 0.48]) and women (AAPC = 0.70%, 95%CI = [0.60, 0.80]), especially in women. ASMR of AD attributable to HFPG indicated an increasing trend among the entire population (AAPC = 0.09%, 95%CI = [−0.07, 0.25]). ASMR of AD caused by HBMI significantly increased in the cohort (AAPC = 2.35%, 95%CI = [2.21, 2.48]), which was higher in men (AAPC = 2.70%, 95%CI = [2.56, 2.84]) than that in women (AAPC = 2.29%, 95%CI = [2.19, 2.39]).

**TABLE 1:** Age-standardized mortality rate per 100,000 of AD attributable to smoking, HFPG, and HBMI in 1990 and 2019, and its temporal trends from 1990 to 2019.

Sex	Smoking			High fasting plasma glucose			High body-mass index		
	ASMR (1990) (95%UI)	ASMR (2019) (95%UI)	AAPC% (95%CI)	ASMR (1990) (95%UI)	ASMR (2019) (95%UI)	AAPC% (95%CI)	ASMR (1990) (95%UI)	ASMR (2019) (95%UI)	AAPC% (95%CI)
Both	3.07 (0.64∼8.99)	3.50 (0.78∼9.63)	0.47^a^ (0.39∼0.55)	1.63 (0.16∼6.57)	1.71 (0.18∼6.63)	0.09 (−0.07∼0.25)	0.86 (0.07∼3.26)	1.67 (0.22∼5.65)	2.35^a^ (2.21∼2.48)
Male	6.25 (1.36∼18.20)	6.92 (1.51∼19.79)	0.37^a^ (0.25∼0.48)	1.31 (0.11∼5.02)	1.79 (0.16∼7.15)	0.56^a^ (0.38∼-.73)	0.63 (0.04∼2.50)	1.36 (0.11∼4.71)	2.70^a^ (2.56∼2.84)
Female	1.28 (0.25∼3.92)	1.56 (0.34∼4.55)	0.70^a^ (0.60∼0.80)	1.79 (0.15∼7.70)	1.58 (0.14∼6.02)	−0.07 (−0.85∼0.10)	0.97 (0.07∼3.90)	1.85 (0.21∼6.35)	2.29^a^ (2.19∼2.39)

UI, uncertainty interval; ASMR, age-standardized mortality rate; CI, confidential interval; AAPC, average annual percentage change; a statistically significant (*p* < 0.05). Population estimates: the GBD was queried for the mortality per 5-year age group from 1990 to 2019, as well as population estimates for each year (https://ghdx.healthdata.org/record/ihme-data/global-population-forecasts-2017–2100).

### 3.2 ASMR for AD attributable to metabolic risks and smoking

**FIGURE 1:**
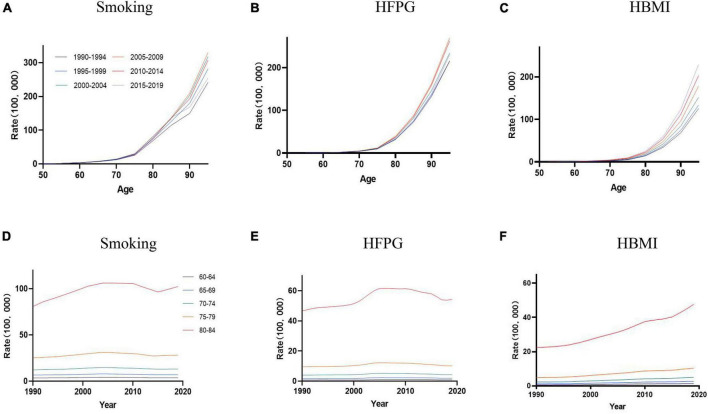
The age-specific mortality rates of AD were attributable to smoking, HFPG, and HBMI in the period between 1990 and 2019, and the cohort-specific mortality rate of AD was attributable to metabolic risks by age group between 1990 and 2019. **(A–C)** Values of the mortality for AD attributable to smoking, HFPG, and HBMI in China from 1990 to 2019. **(D–F)** Survey years were arranged into consecutive 5-year periods from 1990 to 1994 (median, 1992), 1995 to 1999 (median, 1997), 2000 to 2004 (median, 2002), 2005 to 2009 (median, 2007), 2010 to 2014 (median, 2012), and 2015 to 2019 (median, 2017).

Table 1 and Supplementary Figures 1–3 listed the AD mortality attributable to smoking, HFPG, and HBMI by year, age, period, and cohort from 1990 to 2019. AD mortality rises with age; a similar trend is also observed in AD mortality attributable to smoking and HFPG in all periods (Figures 1A–C). From 1990 to 2019, AD mortality attributable to HBMI increased in 50+ age groups (Figure 1C). Mortality caused by smoking in individuals aged 50+ increased from 1990 to 2014, then declined from 2015 to 2019 (Figure 1B). The AD mortality caused by smoking, HFPG, and HBMI showed an increasing trend with age in people over 60 years old, and both reached the highest in the age group of 80–84 (Figures 1D, E).

### 3.3 Net drift and local drift in age groups

During the study period, there was an increase in AD mortality attributable to smoking, as indicated by the overall net drift (%/year) (0.13, 95%CI = [0.05, 0.20]), which was more significant in the net drift for women (0.46, 95%CI = [0.09, 0.82]) than men (−0.03, 95%CI = [−0.11, 0.05]). The net drift values of AD mortality caused by HFPG for men and women were 0.82% (95% CI = [0.60, 1.05]) and 0.43% (95% CI = [0.22, 0.64]), respectively, while those for HBMI were 3.14% (95% CI = [2.89, 3.39]) and 2.76% (95% CI = [2.55, 2.96]), reflecting a substantial increase in AD mortality.

**FIGURE 2:**
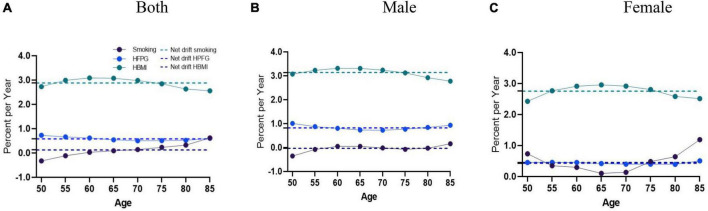
Local drift with net drift values of the mortality for AD attributable to metabolic risks (HFPG and HBMI) in China from 1990 to 2019. **(A)** Corresponding to both sexes; **(B)** corresponding to males; **(C)** corresponding to females. The dots and shaded areas denote percentage and their corresponding 95%CI.

Local drift values of AD mortality caused by HFPG and HBMI were above 0 in 50+ groups; the trend for HFPG showed a stable trend in AD mortality in both men and women, while the trend for HBMI initially enhanced and then reduced in men and women. The most significant decrease was observed in males between the ages of 75–80 (−21.39%%; 95%CI: −24.72∼−18.07) and women aged 75–80 (−20.37%%; 95%CI: −22.84∼−17.9). In contrast, local drift values of AD mortality caused by smoking were partly less than 0 for men, showing an initial increase, followed by a decrease, and then another increase across all ages in men; for women, the trend showed a decrease followed by an increase (Figures 2A–C).

### 3.4 APC effects on AD mortality attributable to metabolic risks and smoking

**FIGURE 3:**
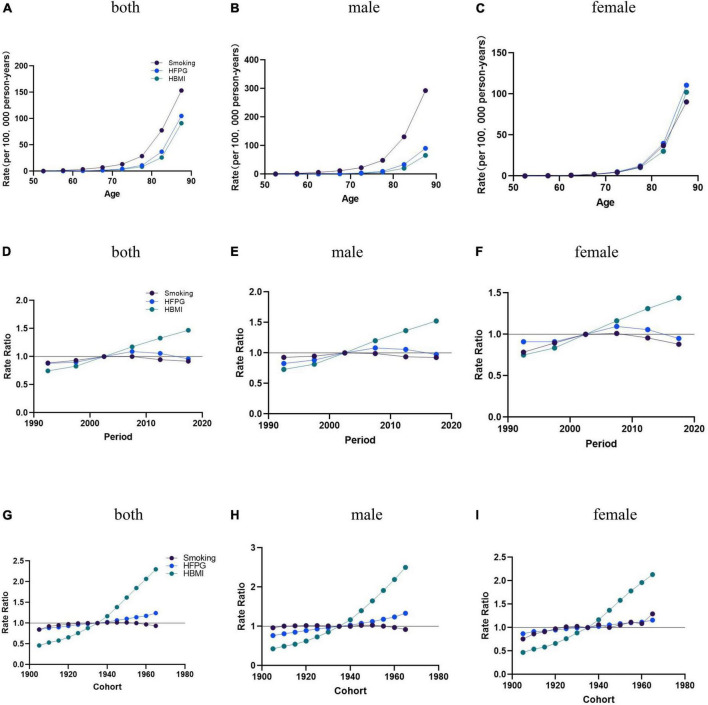
The age-period-cohort (APC) results of AD attributable to smoking, HFPG, and HBMI in China from 1990 to 2019. **(A–C)** Fitted longitudinal age curves of AD mortality (per 100,000) attributable to smoking, HFPG, and HBMI, with **(A)** corresponding to both sexes, **(B)** corresponding to males, **(C)** and corresponding to females. **(D–F)** The rate ratio of each period compared with the reference (2000–2005) adjusted for age and nonlinear cohort effects, with **(D)** corresponding to both sexes, **(E)** corresponding to males, **(F)** and corresponding to females. **(G–I)** The rate ratio of each cohort compared with the reference (cohort 1900–1980) adjusted forage and nonlinear period effects, with **(G)** corresponding to both sexes, corresponding to males, and **(I)** corresponding to females. The dots and shaded areas denote mortality rates or rate ratios.

Age effects on AD mortality caused by smoking, HFPG, and HBMI follow the exponential increase trends, with a significantly accelerated rate observed in individuals aged 80 and above. AD mortality attributable to HBMI in men of all age groups and women aged 50∼70 remained consistently low. Attributable to HBMI, AD mortality in women was more significant than in men aged 70+ (Figures 3A–C).

Age effects: The overall population-wide changes (Net Drift) and 95% confidence intervals for AD mortality attributable to smoking, HFPG, and HBMI in China between 1990 and 2019 were 0.13 (95% CI = [0.05, 0.20]), −0.58 (95% CI = [0.43, 0.73]), and 2.88 (95% CI = [2.72, 3.04]), AD mortality enhanced with age after age 60 years. In the overall and male populations, AD mortality attributable to smoking was significantly higher than the mortality attributable to HFPG and HBMI. In the female population, AD mortality attributable to HFPG slightly outpaced mortality trends attributable to smoking and HBMI (Figures 3A–C).

Period effects: The period effects of AD mortality attributable to HBMI for the overall, men and women all trended upward from 1990 to 2019 in China. Using 2000–2004 as the reference value, the period effect of AD mortality attributable to smoking increased until 2000–2004 and then decreased. The period effects of AD mortality caused by HFPG rose between 1990 and 2019 and have all trended downward since then (Figures 3D–F).

Cohort effects: Birth cohort effects attributable to smoking and HFPG mortality rates remained relatively flat from 1905 to 1950, with a relative increase in mortality rates attributable to HFPG and a decrease in mortality rates attributable to smoking after 1950, and a steady upward trend in the period effects of AD mortality rates attributable to HBMI for the overall, male, and female populations, with a more pronounced trend of growth over time (Figures 3G–I).

## 4 Discussion

This study thoroughly evaluated the temporal trends in AD mortality attributable to smoking and metabolic risks (HFPG and HBMI). The number of AD deaths rose by 289.37% in 2019 compared with 1990, with the mortality rate rising by 233.65% and the standardized mortality rate rising by 20.62%. The study indicated that the AD mortality and standardized mortality rates attributable to smoking, HFPG, and HBMI in China demonstrated a consistent upward trajectory between 1990 and 2019, which may be attributed to the increasing population of older individuals in China. As of the conclusion of 2023, the total number of individuals who were 60 years old or older amounted to 296.97 million, representing 21.1% of the country’s entire population. In China, the AD deaths number caused by smoking in 2019 increased by 251.86% from 1990, the mortality rate increased by 193.23%, and the standardized mortality rate increased by 14.01%. The AD deaths number caused by HFPG in 2019 increased by 267.86% from 1990, the mortality increased by 207.27%, and the standardized mortality increased by 4.27%. The AD deaths number attributable to HBMI in China in 2019 increased by 573.66% from 1990, mortality increased by 456.67%, and standardized mortality increased by 94.19%. Age is an essential factor in AD mortality, and the results showed that the AD mortality rate attributable to smoking, HFPG, and HBMI in China increased with age, and it was most pronounced, especially in the population aged 60+ years.

Nevertheless, the increased mortality linked with AD is not just attributed to life expectancy but is also influenced by an increase in premorbid diagnosis or identification (Zhao et al., 2021). In recent years, AD’s diagnostic efficiency and classification accuracy have been improved due to advancements in artificial intelligence and neuroimaging, and the detection rate has been increasing (Scheltens et al., 2021). Unfortunately, due to the many clinical characteristics and intricate pathology classifications of AD contributing to its variability, there is no effective treatment, and the available treatments only slow down the progression of AD (Lee et al., 2019). The large base of AD patients, high diagnosis rate, and difficulty of cure lead to a cohort effect presenting an elevated trend in AD mortality. Early identification of AD can potentially improve clinical outcomes and enhance the quality of life for both patients and caregivers, although it may not affect the course of the disease or the rate at which it progresses (Sano, 2004; Kokkinou et al., 2021). Due to neuropathologic changes in AD patients occurring insidiously before diagnosis, early and accurate prediction of high-risk factor populations is needed (Kivimäki et al., 2023). Cardiovascular health is directly related to brain health or cerebrovascular health. Since the brain relies heavily on arteries to transport blood, oxygen, and nutrients and to remove waste and toxins, it is vital to have healthy arteries in the brain. When these arteries are damaged, narrowed by blockages of fat and cholesterol, or even completely blocked, the brain is unable to get the vital substances it needs to function, which damages the brain and weakens its ability to fight off infection and disease processes, and can even lead to dementia if specific areas of the brain are sufficiently damaged. With age, the production of cerebrospinal fluid decreases, a decline exacerbated by AD, and people are increasingly exposed to risk factors for HFPG and HBMI. The latest recommendations suggest that a brain-healthy diet that reduces smoking behavior reduces the risk of heart disease and diabetes and is low in saturated fat and cholesterol, which can help protect the brain from disease. Lifestyle factors such as smoking and diabetes may exacerbate AD, and changing lifestyle is one of the comprehensive ways to treat AD. In addition, doctors can accurately classify patients according to their disease risk. High-risk patients will begin treatment with risk reduction, such as medication, weight loss, exercise, and lifestyle changes. Early detection, early intervention, use of appropriate medication, and comprehensive lifestyle adjustments can all dramatically reduce morbidity and mortality in individuals who follow treatment regimens (Sahyouni et al., 2021). This combination of treatments for the disease is an ideal way to avoid the harmful consequences of cardiovascular disease and is a similar treatment that could treat AD.

The mortality of risk factors for AD has significantly increased, including smoking, HFPG, and HBMI; the risk factors can potentially interact with the birth cohort and lead to an increased death rate in individuals with AD. On average, older adults born later in the cohort had higher education levels. The rising levels of education among older individuals during the previous 15 years may have influenced the outcomes of dementia and mortality (Langa et al., 2008). Increased levels of education are thought to be linked to a higher “cognitive reserve”, meaning that the brains of individuals with more education may endure more damage (such as AD pathology or reduced blood flow) before reaching the point of clinically noticeable impairment (Scarmeas et al., 2006; Stern, 2006). Therefore, those with higher levels of education exhibit more advanced brain pathology, leading to a faster deterioration in cognitive function and a higher likelihood of AD death (Cagney and Lauderdale, 2002).

AD mortality caused by HBMI has significantly increased in both, especially in women. HBMI had a more significant effect on the female population in both the period effect and the age effect, suggesting that the combination of smoking and HBMI may have contributed to the birth cohort effect (Jones et al., 2023). Although age is the primary factor in the probability of fatalities caused by AD, lifestyle choices and health circumstances specific to various genders can also influence AD mortality (Health and Retirement Study, 2023; Dhana et al., 2024). The disparities could be attributed to the physiological distinctions inherent in men and women (Ballard et al., 2011). Decision makers could prioritize gender disparities in AD attributable to HBMI and develop appropriate measures to decrease AD mortality. Due to the influence of social culture and living habits, men are more likely than women to be exposed to drinking, high-calorie diet, smoking, and other risk factors leading to HBMI, resulting in a higher BMI growth rate than women, significantly increasing the risk of death from AD. Among the aging population in China, elderly care institutions can strictly control the diet of AD patients, publicize and increase the supply of unsaturated fatty acid food, reduce saturated fatty acid intake, and guide the elderly population to develop better eating habits (Critselis and Panagiotakos, 2020). Women had a higher likelihood of mortality due to AD, while there is no notable connection in men. Therefore, women become a critical intervention group for senile dementia, and health education for male obesity groups should be paid attention to in order to reduce the risk of senile dementia and disease damage. China should strengthen the publicity and education of HBMI on the risk of senile dementia, attach importance to and support the whole population, all-around and life-cycle weight health management, and provide personalized weight loss and cognitive function training services for high-risk groups.

The cohort effect showed that the standardized AD mortality rate attributed to smoking and HFPG increased more slowly compared to the overall AD mortality rate, suggesting that there is still a need to enhance the regulation of smoking habits and diabetes mellitus. The standardized AD mortality rate attributed to HBMI increased more greatly, with a more significant effect on the female population, suggesting that obesity and the level of health have a more remarkable influence on AD mortality and that corresponding health management measures should be developed in a targeted manner.

Time effects showed that AD mortality caused by smoking in China increased from 1990 to 2005 and then decreased from 2005 to 2020, caused by the World Health Organization Framework Convention on Tobacco Control (GBD 2015 Tobacco Collaborators, 2017) officially approved in China on 11 October 2005. China will enter the deep aging society around 2035, facing the challenge of rapid aging and the overlapping risk of death from smoking-induced diseases. Han and Zhou (2015) noted that the severity of the anti-smoking policy positively correlates with the severity of the aging population. AD mortality attributable to smoking has been slightly decreasing in the last several years, which may be attributed to the fact that China has gradually emphasized tobacco control actions and introduced tobacco control measures at the local level. From the perspective of tobacco control, the price elasticity of tobacco products can be used to judge the impact on tobacco consumption. As the price of tobacco products increases, consumers may begin to reassess the impact of smoking on their health and finances, resulting in a reduction in nicotine intake or cessation of smoking. The introduction of excise taxes could therefore provide an economic incentive for tobacco consumers to reduce or stop smoking. It is generally accepted that better management of smoking is already a public health goal for reducing AD mortality, however, many studies overlooked the adverse effects of smoking on women. AD mortality attributable to smoking has decreased slightly in the overall and male populations, which may be due to effective interventions of tobacco control measures in China. Conversely, our study concludes that smoking has had an impact on AD mortality in older women in recent years, it has shown a slight upward trend in the female population. The older female population is a high-risk group for the burden of attribution to smoking, which causes platelet clumping in the brain and thus predisposes to vascular dementia. Older women are more susceptible to AD deaths attributable to smoking behaviors as they age (Doll and Peto, 1981). Older women are affected by the cross-influence of HBMI and smoking risk factors, leading to the aggravation of AD. Shivpuri et al. (2012) and Ishii et al. (2012) noted that women participants exhibited a higher vulnerability to inflammation, potentially rendering them more prone to developing diabetes and metabolic syndrome. Typically, those who smoke tend to have a lower BMI than individuals who do not smoke when matched for age. However, when smokers quit smoking, they often experience a weight increase (Pan et al., 2015; Turnquist et al., 2024). Compared to the population of non-smoking women, the population of women who had ever regularly smoked had a notably elevated risk of mortality from a severe illness, and smokers had a lower BMI than age-matched nonsmokers (Chou et al., 2004, Ellulu, 2018). Moreover, AD develops progressively, as indicated by a multitude of research (Vermunt et al., 2019). Globally, women outlive men by an average of 4.5 years (GBD 2021 Demographics Collaborators, 2024). Due to men having a shorter life expectancy, the manifestations of AD may not have been fully evident at the time of the premature demise. However, women experienced a more significant number of AD-related deaths compared to men, leading to a greater burden for them (Zhao et al., 2021). Therefore, tobacco control policies for women should not be ignored. Although the female smoking population is significantly less than the male population, the female smoking population is more negatively affected, which means that with the progress of the times and the development of culture, female smokers have gradually become a group that needs attention and research. Public health policies should pay attention to the influence of smoking factors on the mortality rate of AD in the female population, avoid the phenomenon of weakening the role of female smokers, fill the gaps in the previous tobacco control policies for the whole population and even tend to men, and control smoking behavior, to achieve the purpose of reducing the mortality rate of AD and more beneficial to the health of the whole population.

In addition, subsequent investigations have revealed a notable rise in the likelihood of developing AD due to smoking, particularly in noncarriers of the APOEε4 allele (Ott et al., 1998; Merchant et al., 1999; Aggarwal et al., 2006). The heritability of late-onset AD is significantly high, with estimates ranging from 60 to 80% (Gatz et al., 2006; Sullivan et al., 2019). Risk factors of older adults for AD are worth studying. This study focused on risk variables that can be changed or modified, such as smoking and metabolic risks.

For the first time, we analyzed GBD 2019 data to reveal the temporal trend of death from AD in China that can be attributed to smoking, HFPG, and HBMI. Using the APC model, we calculated the individual impacts of age, period, and cohort on death due to AD. Additionally, the local drift values accurately represented the temporal trend of AD mortality caused by smoking, HFPG, and HBMI in each age group. Differentiating between the drivers of changes in AD mortality in various periods and birth cohorts was made possible by analyzing period and cohort effects. Our work can help track the aims of sustainable development and identify the target populations for prevention and treatment based on adverse trends in AD mortality attributed to smoking, HFPG, and HBMI in certain age groups.

## 5 Limitations

This study used the GBD 2019 data, which could not explore the changes in AD mortality trends attributed to smoking, HFPG, and HBMI in different regions of China. While we considered period and cohort effects, our APC analysis relied on the estimated cross-sectional data from the GBD. Thus, extensive cohort investigations are necessary for validating the hypotheses of this study.

## 6 Conclusion

In this study, we showed that time trends in AD mortality caused by metabolic risks and smoking in China from 1990 to 2019 have consistently risen. Thus, it is essential to prevent being overweight or obese throughout old age; it is crucial to acknowledge the significance of enhancing the elderly population as well, especially females, and the necessity of promoting smoking prevention and control knowledge and promoting healthy lifestyles to reduce the mortality of AD attributed to smoking in China. The results of this study will also help develop and assess the efficacy of AD and treatment and rehabilitation techniques in China.

## Data availability statement

The datasets presented in this study can be found in online repositories. The names of the repository/repositories and accession number(s) can be found below: https://ghdx.healthdata.org/gbd-2019.

## Author contributions

SS: Data curation, Formal analysis, Visualization, Writing – original draft, Writing – review and editing. TZ: Data curation, Methodology, Writing – review and editing. HY: Writing – review and editing. TX: Writing – review and editing. YY: Software, Writing – review and editing. MS: Validation, Writing – review and editing. HL: Project administration, Writing – review and editing. QH: Formal analysis, Writing – review and editing. WW: Data curation, Resources, Writing – original draft. HFY: Project administration, Writing – review and editing. XH: Data curation, Investigation, Writing – review and editing.

## Footnotes

1
https://vizhub.healthdata.org/gbd-results/

2
https://analysistools.cancer.gov/apc/
