# Supplementary material for: Time trends in Alzheimer’s disease mortality attributable to metabolic risks and smoking in China from 1990 to 2019: an age-period-cohort analysis
Source: Front Aging Neurosci. 2024 Jul 3;16:1425577. doi: 10.3389/fnagi.2024.1425577 (PMC11256009; doi:10.3389/fnagi.2024.1425577)
Supplement: Supplementary file 1 [file Data_Sheet_1.docx]

| A Smoking-Both  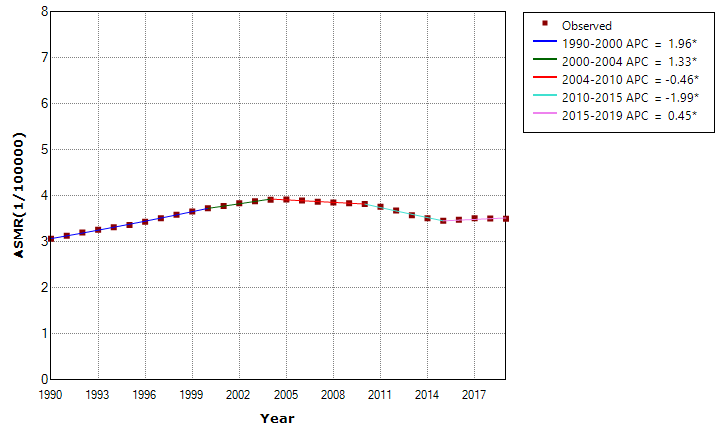 | B Smoking-Male  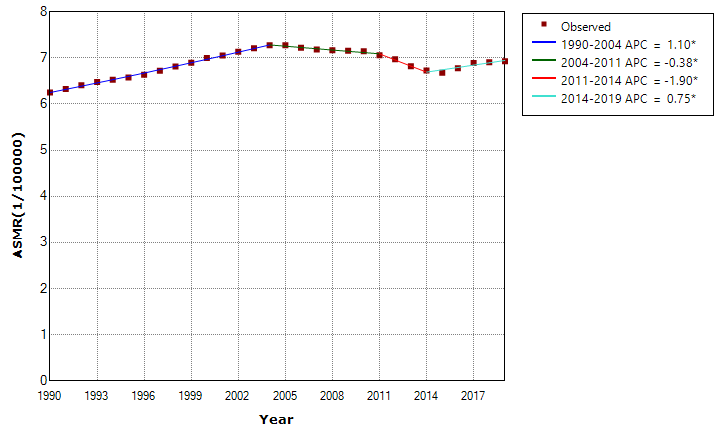 | C Smoking-Female  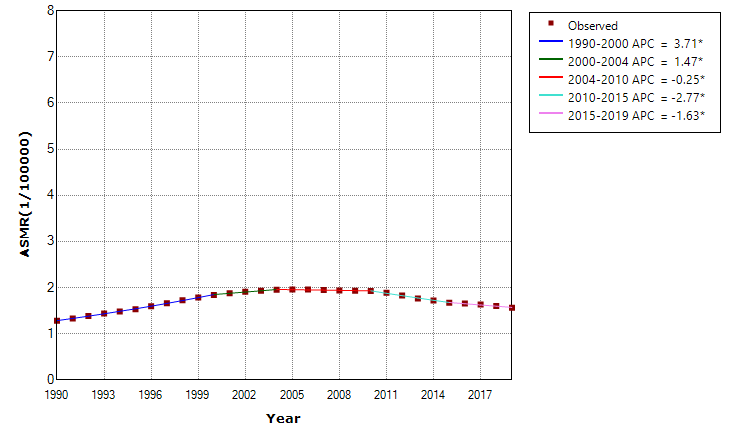 |
| --- | --- | --- |
| D HFPG-Both  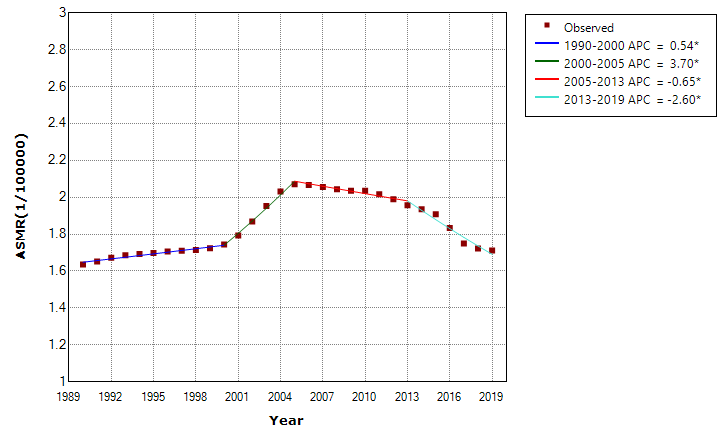 | 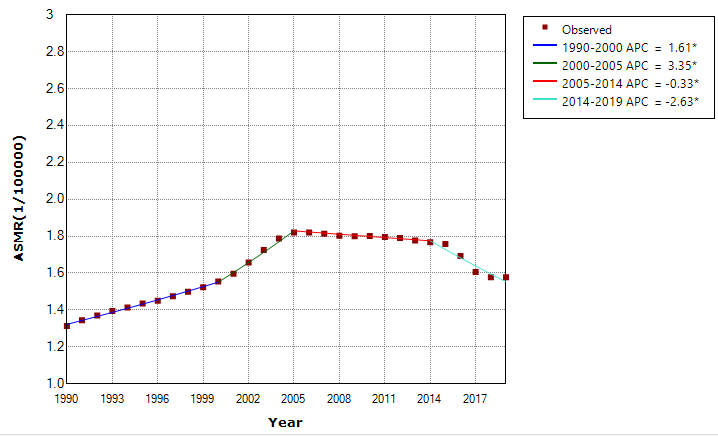E HFPG-Male | F HFPG-Female  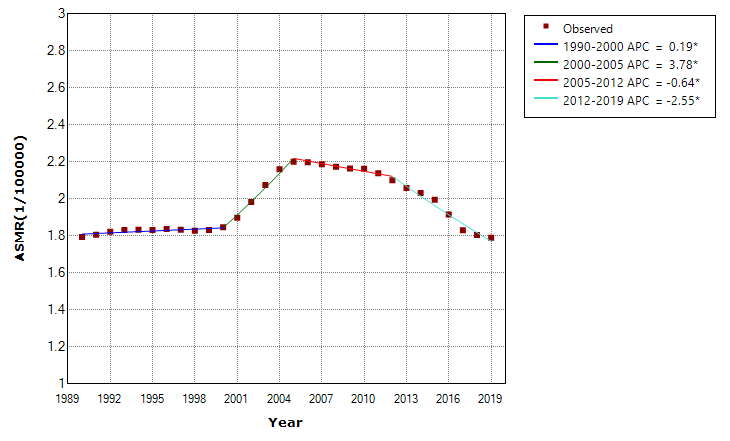 |
| G HBMI-Both  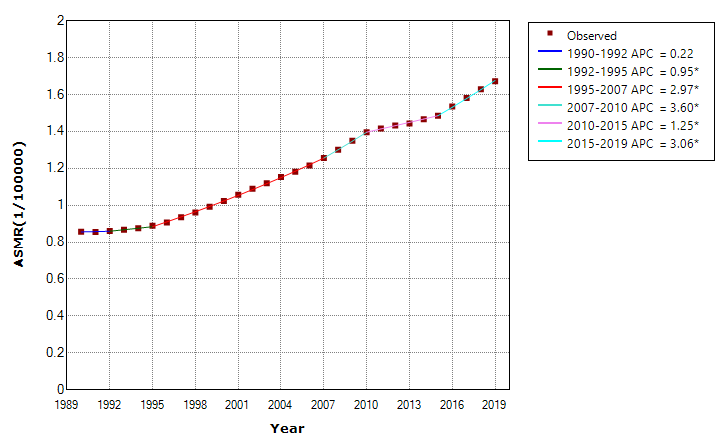 | H HBMI-Male  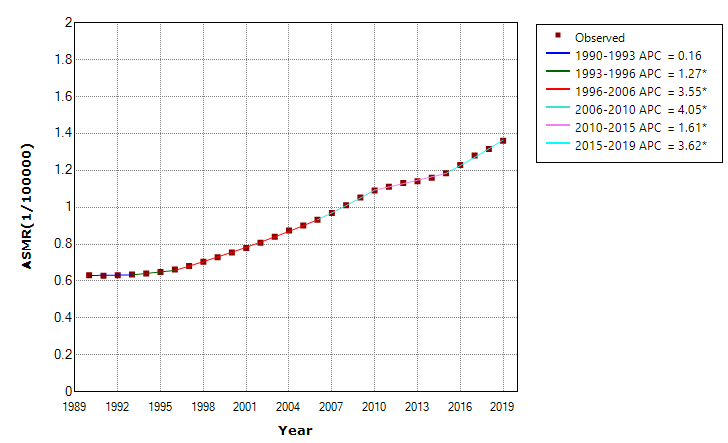 | I HBMI-Female  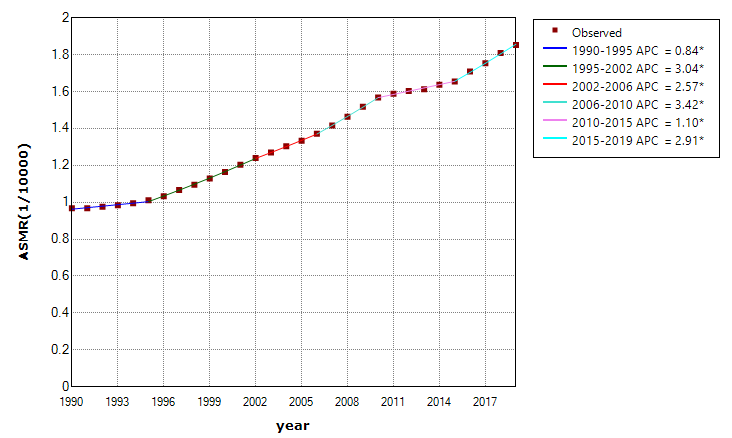 |

**Figure S1.** Joinpiont regression analysis of trends in the ASMR of AD attributable to smoking, HFPG and HBMI in China, 1990 to 2019.(A–C) Fitted Joinpiont regression analysis of trends in the ASMR of AD attributable to smoking, with (A) corresponding to both sexes, (B) corresponding to males, (C) and corresponding to females. (D–F) Fitted Joinpiont regression analysis of trends in the ASMR of AD attributable to HFPG, with (D) corresponding to both sexes, (E)corresponding to males, (F)and corresponding to females. (G–I) Fitted Joinpiont regression analysis of trends in the ASMR of AD attributable to HBMI, with (G) corresponding to both sexes, corresponding to males, and (I) corresponding to females. The dots and shaded areas denote mortality rates or rate ratios.

*Indicates that Annual Percent Change (APC) is significantly different from zero at the alpha=0.5 level

| 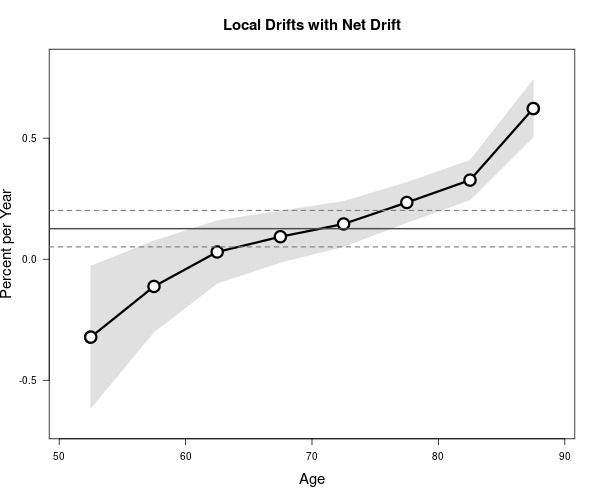A Smoking-Both | 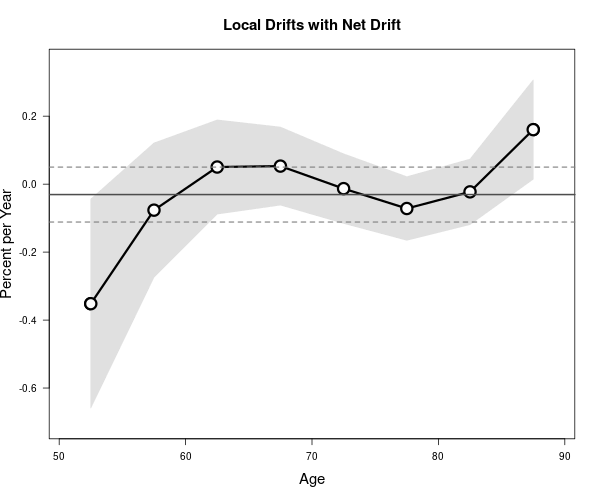B Smoking-male | 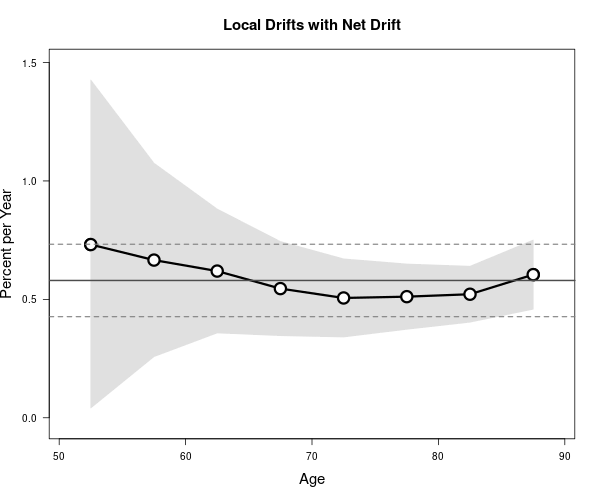C Smoking-female |
| --- | --- | --- |
| D 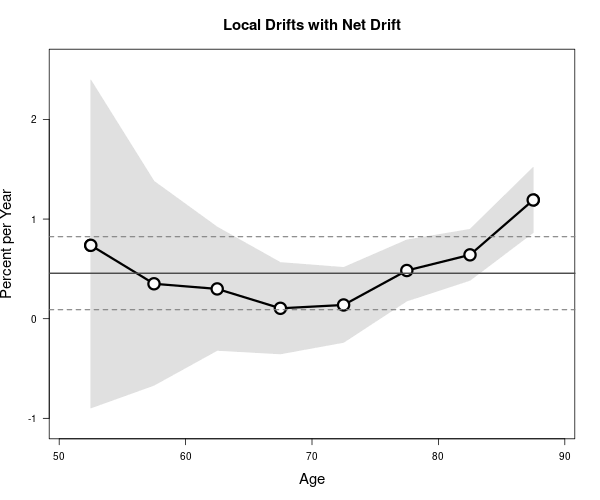HPFG-Both | E 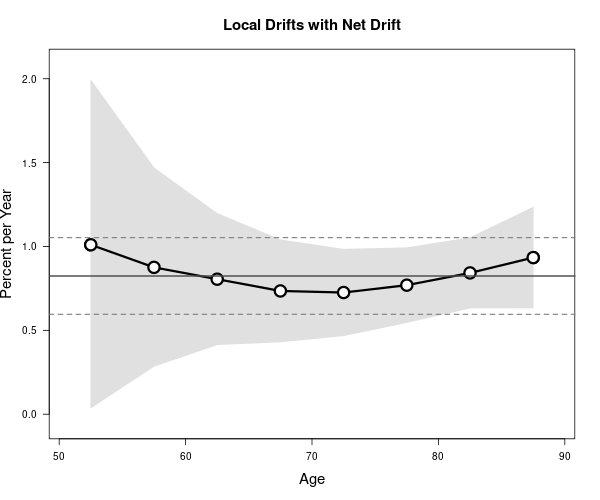HPFG-Male | F 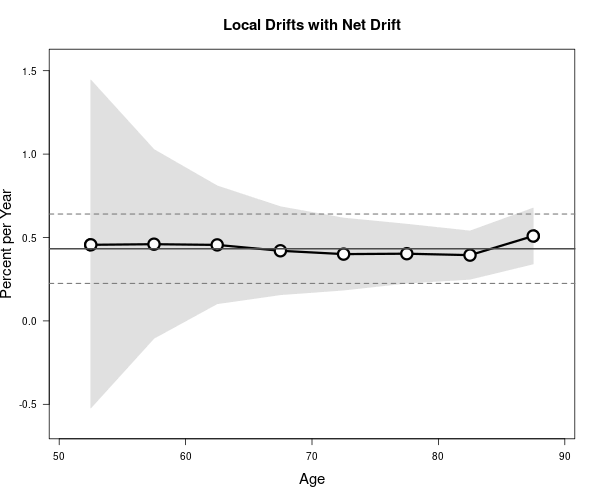HFPG-female |
| G 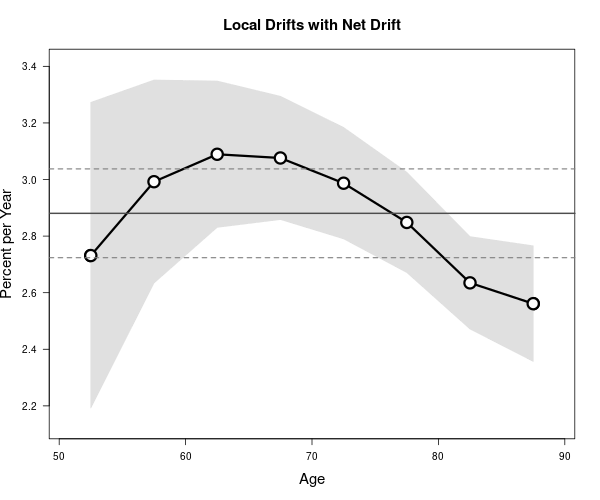HBMI-Both | H 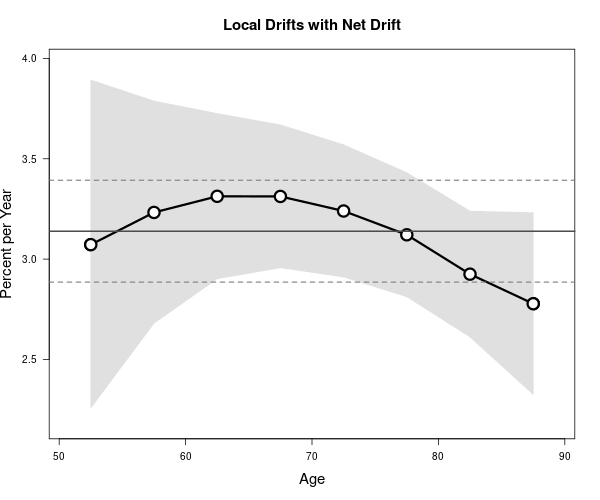HBMI-Male | I 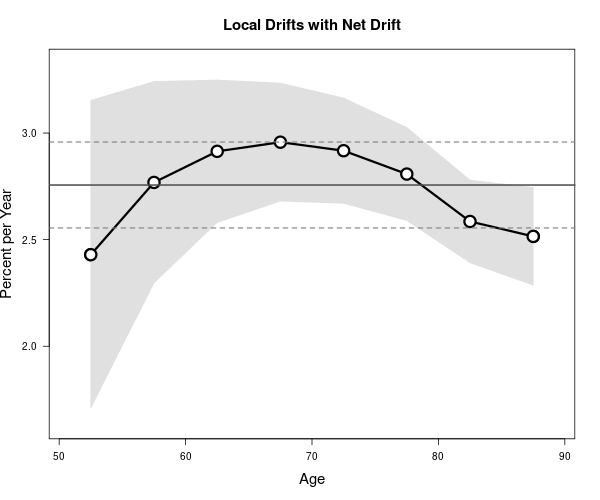HBMI-Female |

**Figure S2.** Trends in local drifts with net drift attributable to smoking, HFPG and HBMI.

**(A-C)** Annual Trends in local drifts with net drift for AD attributable to smoking, 1990-2019.

**(D-F)** Annual Trends in local drifts with net drift for AD attributable to HFPG, 1990-2019.

**(G-I)** Annual Trends in local drifts with net drift for AD attributable to HBMI, 1990-2019.

**
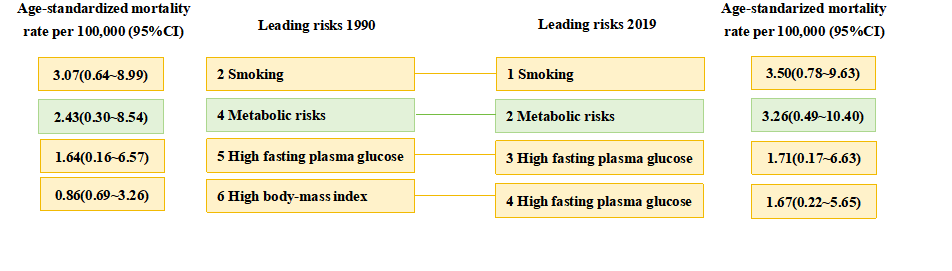
**

**Figure S3.** The rank of all risk factors for AD in China in 1990 and 2019.
